# Comparison of Retention Rates Between Tumor Necrosis Factor-α Inhibitors in Patients With Ankylosing Spondylitis: Data From the Korean College of Rheumatology Biologics Registry

**DOI:** 10.3389/fmed.2021.689609

**Published:** 2021-06-15

**Authors:** Hyoun-Ah Kim, Sun-Kyung Lee, Sohee Oh, Eun Hye Park, Yong-Beom Park, Kichul Shin

**Affiliations:** ^1^Department of Rheumatology, Ajou University Hospital, Suwon, South Korea; ^2^Division of Rheumatology, Seoul Metropolitan Government-Seoul National University Boramae Medical Center, Seoul, South Korea; ^3^Division of Rheumatology, Chung-Ang University Hospital, Seoul, South Korea; ^4^Division of Rheumatology, Yonsei University College of Medicine, Severance Hospital, Seoul, South Korea

**Keywords:** ankylosing spondylitis, drug retention, tumor necrosis factor inhibitors, discontinuation, registry

## Abstract

This study aimed to investigate drug retention rates for various TNF inhibitors (TNFis) commonly prescribed to Korean patients with ankylosing spondylitis (AS) in the Korean College of Rheumatology Biologics registry (KOBIO; December 2012–June 2016). Discontinuation was defined as switching or stopping the biologic agent. Kaplan–Meier curves and Cox's proportional hazard models were used for further analysis. The reasons for discontinuation of TNFis were also assessed. Univariate and multivariate analyses were used to identify possible predictors of discontinuation. Data from 1,005 patients with AS were analyzed with a median follow-up period of 14 months. Seventy-six percent of patients were first-line biologic users. Discontinuation of TNFis occurred in 24.2% (switching in 9.6%) of patients during follow-up. An estimate of the drug failure showed that the adjusted hazard ratio (HR) for golimumab compared to etanercept was 0.441 (95% confidence interval: 0.277–0.703, *p* < 0.001). Reasons for discontinuation included lack of efficacy (32.6%), adverse events (23.6%), clinical improvement (11.2%), and others (32.6%). Predictors of discontinuation using a multivariate analysis were a shorter disease duration (HR: 0.973, *p* = 0.044) and being negative for HLA-B27 (HR: 1.623, *p* = 0.0093). In conclusion, few Korean patients with AS switched to other TNFis during their treatment. The drug retention rate for golimumab was higher than for other agents.

## Introduction

Ankylosing spondylitis (AS) is a chronic inflammatory arthritis that predominantly affects the sacroiliac joint and spine and is characterized by chronic back pain and stiffness (1). It can be associated with peripheral arthritis or enthesitis and extra-articular manifestations, such as uveitis and inflammatory bowel disease. Knowledge of the pathogenesis of AS has led to a number of improvements in its treatment, and in particular the development of biologic agents has been of great benefit. Tumor necrosis factor-α inhibitors (TNFis) are the main therapeutic agents used to treat patients with AS for whom non-steroidal anti-inflammatory drugs are ineffective or are contraindicated. Recently, the interleukin-23/interleukin-17 pathway has been targeted in the treatment of AS. TNFis were, therefore, the only biologic agent available to treat AS prior to the introduction of secukinumab. Seven TNFis are currently approved for the management of AS (etanercept originator, etanercept biosimilars, infliximab originator, infliximab biosimilars, adalimumab, golimumab, and certolizumab). Currently, six TNFis (etanercept originator, etanercept biosimilar, infliximab originator, infliximab biosimilar, adalimumab, and golimumab) are available for the management of AS in Korea. Their efficacy and safety have been demonstrated in several randomized controlled trials (RCTs), open-label studies, and in meta-analyses of AS (1–5). The data showed that all TNFis provide rapid and sustained improvement in disease symptoms and improve function in patients with AS (3–5). Furthermore, several factors, such as a young age, a high baseline level of inflammatory markers, and short disease duration, have been reported to be predictors of a good response to TNFis (6). However, these data are limited in terms of allowing for a direct comparison of the efficacy, safety, and drug survival rate among several TNFis in real-life (7).

Several studies have already been conducted to assess the safety, retention rate, and economic feasibility of using biologic agents, including TNFis, for the treatment of rheumatoid arthritis (RA) in real-life (8–12). A recent Japanese cohort study evaluated the reasons for retention or discontinuation of seven biologic agents in real-life in patients with RA (8). Overall, 43.0% of such agents were stopped, with 20.9% being discontinued due to lack of efficacy, 10.9% due to non-toxicity related, 8.3% due to toxic adverse events, and 3.8% due to disease remission. Another study has reported the 12-year retention rate of initial TNFis treatments in a cohort of RA patients (10). The overall 12-year drug retention rate was only 23.4%, and was significantly higher for etanercept than for infliximab and adalimumab. One study analyzed and compared the long-term retention rate of TNFis when used to treat spondyloarthritis (SpA), which included patients with AS and psoriatic arthritis (PsA), and RA in clinical practice (13). They showed that the overall 10-year retention rate of a first-line TNFis was ~23% and that the retention rates for patients with SpA were significantly higher than for patients with RA.

Although the retention rates for TNFis tend to be higher in patients with AS than for patients with RA, there are few studies examining Asian patients with AS in the literature. The aim of this study was to analyze the drug retention rates of TNFis as well as to explore the reasons for their discontinuation in Korean patients with AS.

## Methods

### Study Population

The Korean College of Rheumatology Biologics Registry (KOBIO) was designed as a nationwide multicenter, hospital-based observation registry by the Korean College of Rheumatology as previously described (14–16). KOBIO was established to evaluate the effectiveness and adverse events related to the use of biologic agents in Korean patients with RA, AS, and PsA. The KOBIO registry enrolls patients initiating a new biologic agent, or switching to another biologic agent. Standardized protocols for patient enrollment and data quality control are monitored by all participating centers. Data are collected at enrollment and approximately every year thereafter. This study is a retrospective analysis of prospectively collected data. For this study, subjects were identified using the baseline data of patients with AS who enrolled in the KOBIO and were required to meet the modified New York criteria for AS (17). In total, 1,005 patients with AS were enrolled in the KOBIO registry from December 2012 to June 2016. This study was approved by the Institutional Review Board of each participating hospital and informed consent was obtained from all enrolled patients.

All patient data were input into the KOBIO web server (http://www.rheum.or.kr/kobio/) by individual investigators. For this study, the following AS data were obtained from the KOBIO web server: patient age, sex, body mass index (BMI), disease duration, smoking status, and family history of SpA. BMI was calculated by dividing weight (kg) by height squared (m^2^). The positivity for human leukocyte antigen B27 (HLA-B27) was recorded. Disease activity indexes included the Bath Ankylosing Spondylitis Disease Index (BASDAI), Bath Ankylosing Spondylitis Functional Index (BASFI), C-reactive protein (CRP) levels, and AS disease activity score (ASDAS). All approved and commonly prescribed TNFis were included in the analysis: etanercept, infliximab originator, infliximab biosimilar, adalimumab, and golimumab, either as first-line treatment or following a change from another TNFi. Patients previously treated with biologic therapies prior to screening may be included; however, the efficacy outcome of this previous treatment is not recorded. Treatment-related data included details of the treatment received (dates and line of therapy). Treatment changes and reasons for changing, and discontinuations and reasons for discontinuation were recorded. Adverse events, disease assessment, and medications were documented at the initial and subsequent visits. Discontinuation was defined as switching or stopping the biologic agent. Reasons for discontinuation were classified as: (1) significant clinical improvement, (2) lack of efficacy, (3) adverse events, and (4) other reasons including loss to follow-up, planned or confirmed pregnancy, and cost or reimbursement issues. Patients who did not have a documented time of discontinuation were excluded from this analysis. If patients were recorded as first-line users and their subsequent line of treatment involved a change in the prescribed TNFi due to the failure of the first drug, the data for these patients were censored after the change in prescription and included only in the group of first-line treatment.

### Statistical Analysis

Baseline data at the time of registry enrollment, and follow-up data collected every year thereafter, were used for the analysis. Kaplan–Meier curves were constructed to determine drug retention rates and time to discontinuation of treatment. Cox's proportional hazards model with adjustment for covariates including age, sex, and disease duration was used to compare the time to discontinuation of golimumab treatment with that of other TNFis. The reasons for TNFi discontinuation were also assessed. Univariate and multivariate analyses were used to identify possible predictors of discontinuation. Confounders including age and sex were considered for multivariate analysis to identify the predictors of drug discontinuation. Statistical analyses were performed using SPSS software (ver. 22.0; SPSS Inc., Chicago, IL, USA). A *p* < 0.05 was regarded as indicative of statistical significance.

## Results

### Baseline Clinical Characteristics of the Study Patients

Table 1 shows the baseline clinical characteristics of the study patients. Data from 1,005 patients with AS were analyzed. The mean patient age was 40.7 years, and 77.4% were male. The mean disease duration since the initial diagnosis of AS was 7.1 ± 5.9 years, and the mean BMI was 23.4 ± 3.4 kg/m^2^. A total of 82.4% of patients were found to be positive for HLA-B27 and 33.2% of patients had syndesmophyte lesion(s). The mean baseline BASDAI was 6.0 ± 2.0 and the mean BASFI was 3.5 ± 2.6. The mean ASDAS-CRP level was 3.6 ± 1.1. In total, 76% of patients were first-line biologic users, 17.9% were second-time users, and 6.1% were third-time or more users. Adalimumab was the most commonly prescribed biologic agent (*n* = 375, 37.3%), followed by golimumab (*n* = 225, 22.4%), infliximab biosimilar (*n* = 166, 16.5%), etanercept (*n* = 148, 14.7%), and infliximab originator (*n* = 91, 9.1%). There were no significant differences in clinical characteristics among the patients based on the particular TNFis prescribed.

**Table 1:** Baseline characteristics of the study patients.

	**Etanercept**	**Infliximab originator**	**Infliximab biosimilar**	**Adalimumab**	**Golimumab**	**Total**
*N*	148	91	166	375	225	1,005
Age (years)	41.2 ± 12.8	43.0 ± 14.8	41.1 ± 12.6	40.1 ± 13.0	40.3 ± 10.8	40.7 ± 12.6
Gender, male, *N* (%)	125 (84.5)	60 (65.9)	119 (71.7)	293 (78.1)	181 (80.4)	778 (77.4)
BMI	23.4 ± 3.9	22.9 ± 3.2	23.9 ± 3.1	23.3 ± 3.6	23.2 ± 3.2	23.4 ± 3.4
Disease duration, years	7.7 ± 6.4	6.6 ± 5.3	6.1 ± 5.5	6.8 ± 5.6	8.3 ± 6.5	7.1 ± 5.9
Cigarette smoking, *n* (%)						
Ex	37 (25.0)	18 (19.8)	32 (19.3)	77 (20.5)	46 (20.4)	210 (20.9)
Current	50 (33.8)	25 (27.5)	45 (27.1)	110 (29.3)	72 (32.0)	302 (30.0)
Never	61 (41.2)	48 (52.7)	89 (53.6)	188 (50.1)	107 (47.6)	493 (49.1)
HLA-B27 positivity, %	86.5	86.8	75.3	85.9	77.3	82.4
Family history for SpA, %	12.2	13.2	12.7	7.2	13.3	10.8
BASDAI	6.0 ± 2.0	5.4 ± 2.0	6.2 ± 1.8	5.8 ± 2.0	6.3 ± 1.8	6.0 ± 2.0
BASFI	3.5 ± 2.6	2.9 ± 2.5	3.5 ± 2.4	3.5 ± 2.7	3.6 ± 2.5	3.5 ± 2.6
CRP, mg/dL	2.6 ± 3.2	2.1 ± 3.3	2.1 ± 2.6	2.1 ± 2.7	2.1 ± 2.4	2.2 ± 2.7
ASDAS-CRP	3.7 ± 1.1	3.1 ± 1.2	3.7 ± 1.0	3.5 ± 1.1	3.7 ± 1.0	3.6 ± 1.1
Patients with syndesmophyte, %	33.1	30.8	33.7	30.9	37.8	33.2
Biologic use, *N* (%)						
First-line users	113 (76.4)	61 (67.0)	144 (86.8)	291 (77.6)	155 (68.9)	764 (76.0)
Second-line users	30 (20.3)	26 (28.6)	17 (10.2)	71 (18.9)	36 (16.0)	180 (17.9)
Third or more-line users	5 (3.4)	4 (4.4)	5 (3.0)	13 (3.5)	34 (15.1)	61 (6.1)

*BMI, body mass index; HLA, human leukocyte antigen; SpA, spondyloarthopathy; BASDAI, Bath Ankylosing Spondylitis Disease Index; Bath Ankylosing Spondylitis Functional Index; CRP, C-reactive protein; ASDAS, ankylosing spondylitis disease activity score*.

### TNFi Drug Retention

**Figure 1:**
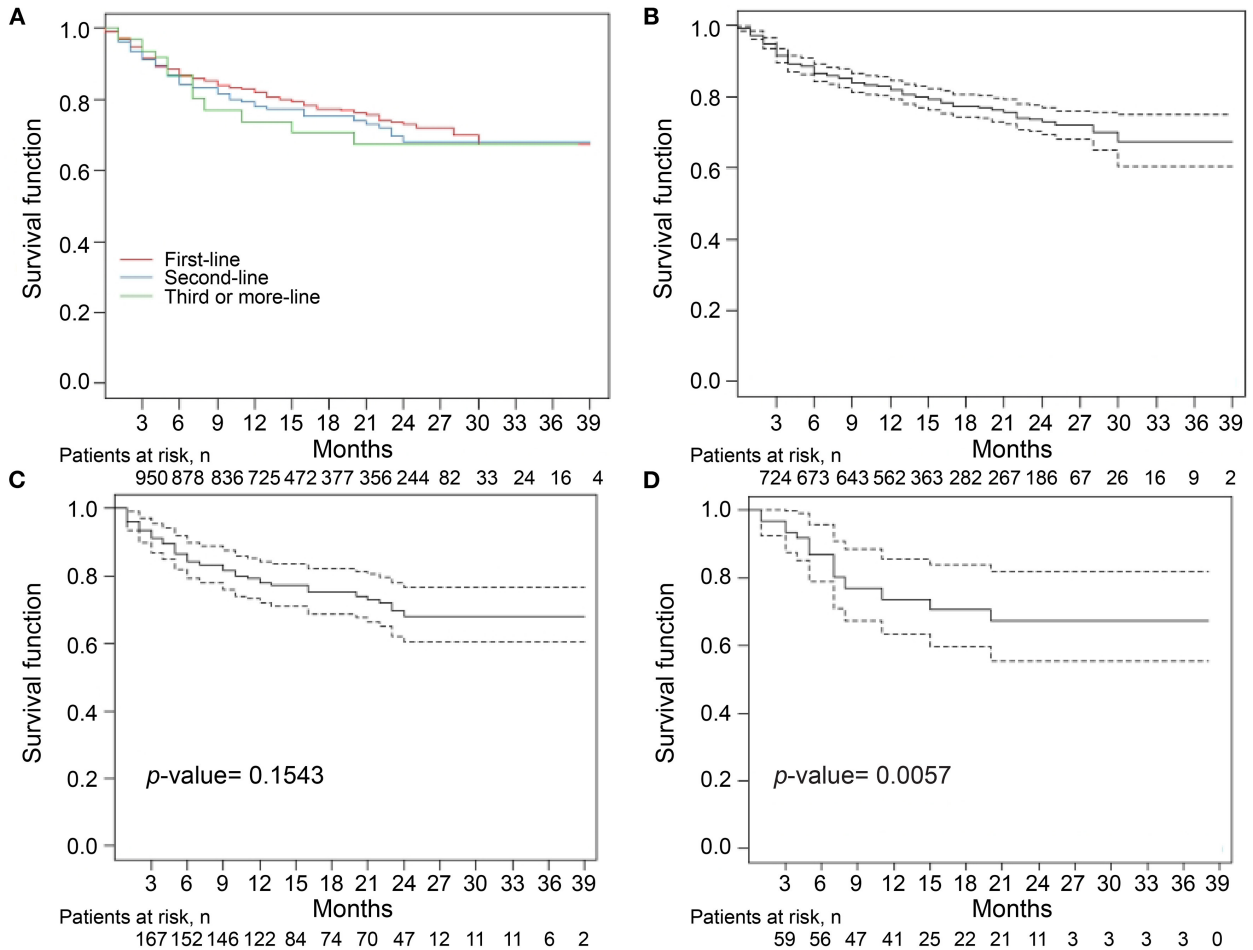
Drug retention rates for tumor necrosis factor inhibitors (TNFi) in patients with ankylosing spondylitis (AS) **(A)**. The median follow-up period was 14 months. The overall drug retention rate for TNFi was 75.8%. Estimate of the drug survival function showed that the adjusted hazard ratio (HR) for third-time biologic users **(D)** compared with first-time biologic users **(B)** was 2.104 (95% CI: 1.241–3.566, *p* = 0.0057), whereas the adjusted HR for second-time biologic users **(C)** compared with first-time biologic users was 1.2738 (95% CI: 0.913–1.777, *p* = 0.1543). Kaplan–Meier curve were conducted for drug retention and time to treatment discontinuation. A Cox proportional hazard model with adjustment for covariates including age, sex, and disease duration was used to compare the time to discontinuation for third-time biologic users with that for first-time biologic users.

**Figure 2:**
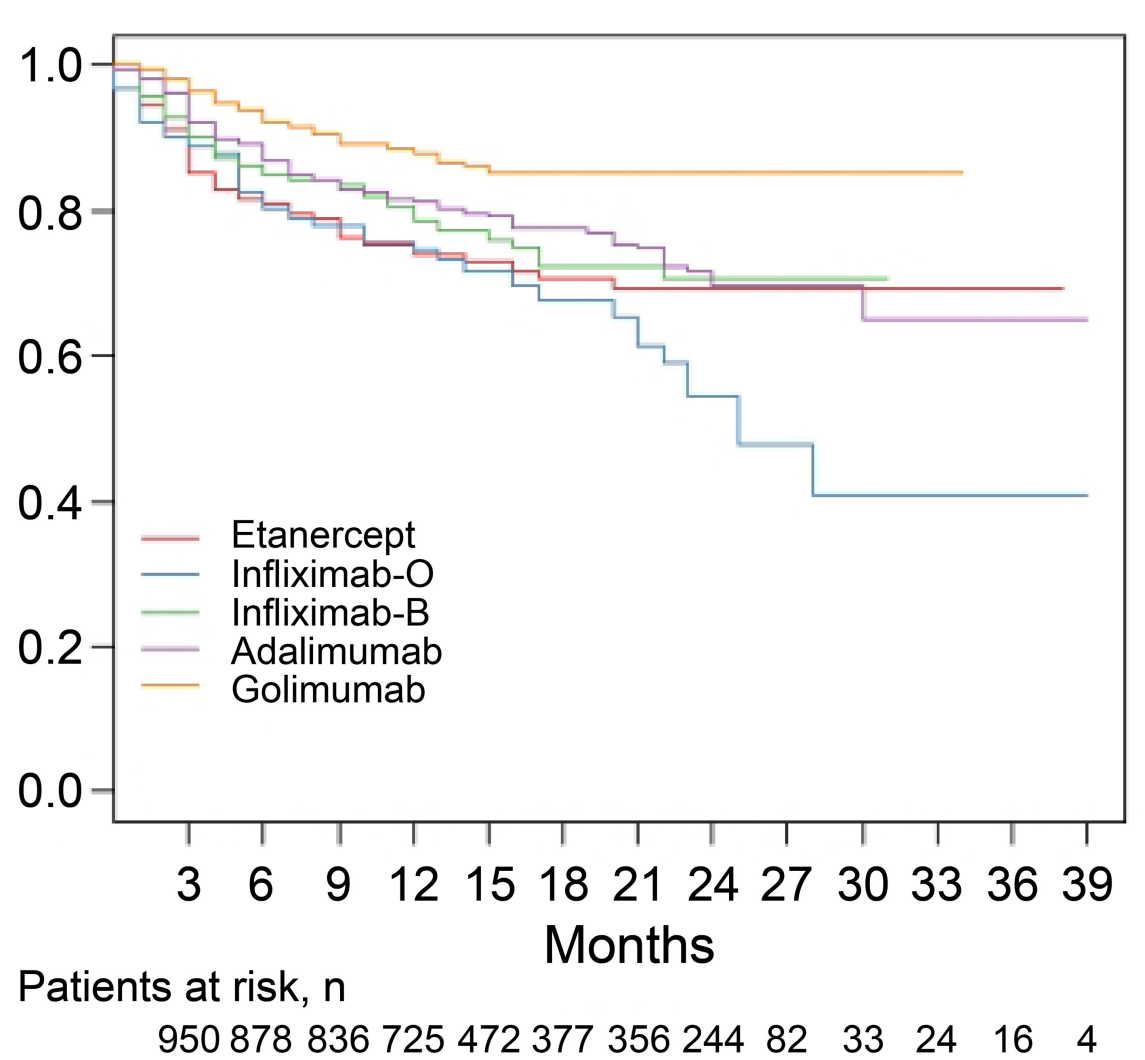
Drug retention rates for individual tumor necrosis factor inhibitors (TNFi) in patients with ankylosing spondylitis (AS). An estimate of the drug failure showed that the adjusted hazard ratio (HR) for golimumab compared to etanercept was 0.441 (95% CI: 0.277–0.703, *p* < 0.001). A Cox proportional hazard model with adjustment for covariates including age, sex, and disease duration was applied to compare the time to discontinuation of golimumab treatment with the time to discontinuation of other TNFis. O, originator; B, biosimilar.

The median follow-up period was 14 months (Table 2). The overall drug retention rate for all TNFis was 75.8% (Figure 1). Estimates of drug survival function showed that the adjusted hazard ratio (HR) for third-line or more biologic users (compared to first-time users) was 2.104 [95% confidence interval (CI): 1.241–3.566, *p* = 0.0057], while the adjusted HR for second-line biologic users (compared to first-line users) was 1.2738 (95% CI: 0.913–1.777, *p* = 0.1543). The drug retention rate for golimumab (82.2%) was highest, followed by adalimumab (75.5%), infliximab biosimilar (74.7%), etanercept (71.6%), and infliximab originator (60.4%). An estimate of the drug failure showed that the adjusted hazard ratio (HR) for golimumab compared to etanercept was 0.441 (95% CI: 0.277–0.703, *p* < 0.001, Figure 2).

**Table 2:** Retention rate of individual tumor necrosis factor inhibitors.

	**Etanercept**	**Infliximab originator**	**Infliximab biosimilar**	**Adalimumab**	**Golimumab**	**Total**
	**(*n* = 148)**	**(*n* = 91)**	**(*n* = 166)**	**(*n* = 375)**	**(*n* = 225)**	**(*n* = 1,005)**
Discontinued, *n*	42	36	42	92	31	243
Discontinuation rate, %	28.4	39.6	25.3	24.5	13.8	24.2
Switched, *n*	17	15	24	31	9	96
Switching rate, %	11.5	16.5	14.5	8.3	4.0	9.6
Follow-up period, months						
Minimum	0.7	0	0.5	0.4	1	0
Maximum	38	39	31	39	34	39
Mean	15.5	15.5	15.1	16.0	16.5	15.9
Median	14	14	13	14	15	14

Table 2 shows the discontinuation and follow-up duration for the total and individual TNFis. Discontinuation of TNFis occurred in 24.2% (*n* = 243) of patients during follow-up, with 14.6% stopping the TNFis completely and 9.6% switching to another TNFis. Discontinuation occurred in 13.8% of golimumab users (stopping: 9.8%, switching: 4.0%), 39.6% of infliximab originator users (stopping: 23.1%, switching: 16.5%), 28.4% of etanercept users (stopping: 16.9%, switching: 11.5%), 25.3% of infliximab biosimilar users (stopping: 10.8%, switching: 14.5%), and 24.5% of adalimumab users (stopping: 16.2%, switching: 8.3%). The median follow-up period for each group was similar, at 13~15 months.

### Reasons for Discontinuation of TNFis

Reasons for discontinuation of TNFis are listed in Table 3. Reasons given for discontinuation included lack of efficacy (32.6%), adverse events (23.6%), clinical improvement (11.2%), and others (32.6%). Reasons for the discontinuation of etanercept were lack of efficacy (*n* = 14, 33.3%), adverse events (*n* = 14, 33.3%), clinical improvement (*n* = 3, 7.1%), and others (*n* = 11, 26.2%). Reasons for the discontinuation of infliximab originator were lack of efficacy (*n* = 10, 27.8%), adverse events (*n* = 12, 33.3%), clinical improvement (*n* = 3, 8.3%), and others (*n* = 11, 30.6%). The most common reason for discontinuing infliximab biosimilar was lack of efficacy (45.2%), followed by adverse events (21.4%), and other reasons (19.1%). Reasons for the discontinuation of adalimumab were lack of efficacy (28.3%), adverse events (20.7%), clinical improvement (15.2%), and others (35.9%). The most common reason for discontinuing golimumab was lack of efficacy (*n* = 10, 32.3%), followed by other reasons (*n* = 16, 51.6%), adverse events (*n* = 3, 9.7%), and clinical improvement (*n* = 2, 6.5%).

**Table 3:** Reasons for discontinuation.

	**Total**	**Etanercept**	**Infliximab originator**	**Infliximab biosimilar**	**Adalimumab**	**Golimumab**
*N*	1,005	148	91	166	375	225
Discontinuation, *n* (%)	243 (24.2)	42 (28.4)	36 (39.6)	42 (25.3)	92 (24.5)	31 (13.8)
Significant clinical improvement, *n* (%)	27 (11.2)	3 (7.14)	3 (8.3)	5 (11.9)	14 (15.2)	2 (6.5)
Inefficacy, *n* (%)	79 (32.6)	14 (33.3)	10 (27.8)	19 (45.2)	26 (28.3)	10 (32.3)
Adverse event, *n* (%)	57 (23.6)	14 (33.3)	12 (33.3)	9 (21.4)	19 (20.7)	3 (9.7)
Other reason^*^, *n* (%)	80 (32.6)	11 (26.2)	11 (30.6)	8 (19.1)	33 (35.9)	16 (51.6)

* *Includes follow-up loss, planned or confirmed pregnancy, costs or reimbursement issues*.

**Figure 3:**
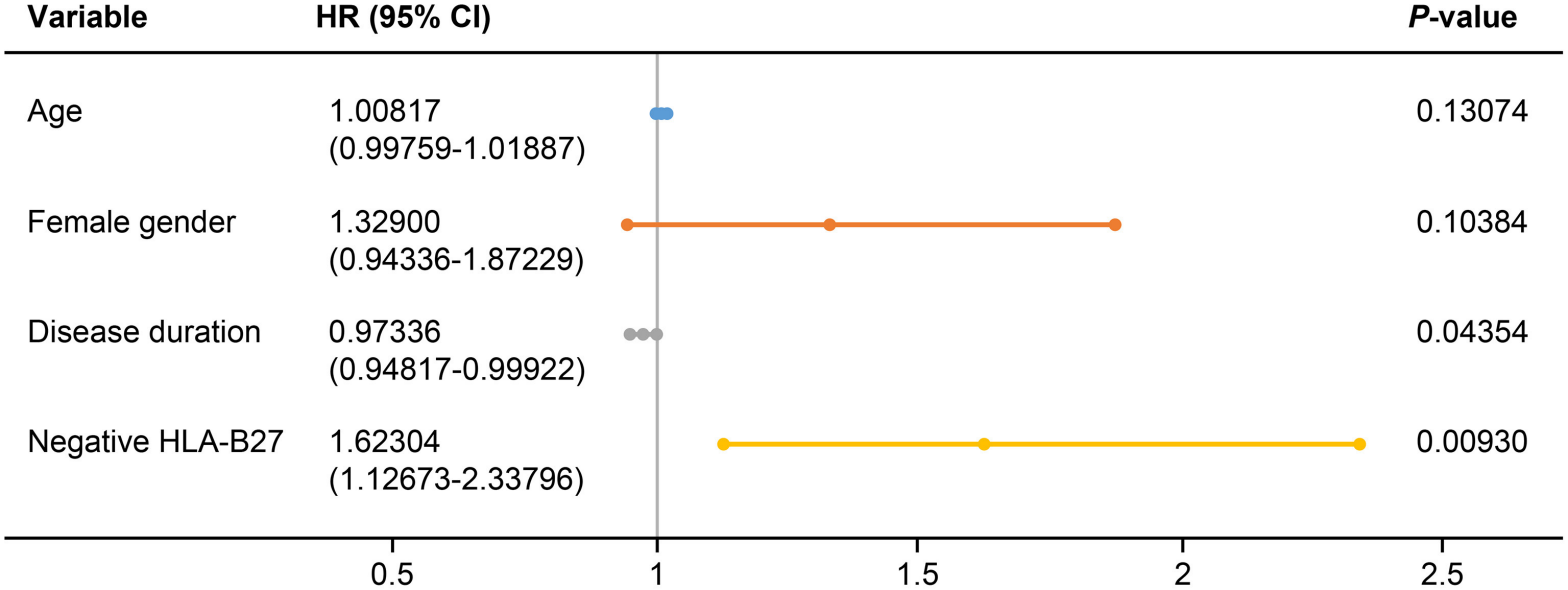
Forest plot based on the results of multivariate analyses of the factors associated with discontinuation of TNFis. HLA, human leukocyte antigen; HR, hazard ratio; CI, confidence interval.

We evaluated predictors associated with the discontinuation of TNFis (Table 4). Age (HR: 1.010, 95% CI: 1.001–1.020, *p* = 0.03851), female sex (HR: 1.525, 95% CI: 1.156–2.011, *p* = 0.00281), disease duration (HR: 0.974, 95% CI: 0.951–0.998, *p* = 0.03253), and being negative for HLA-B27 (HR: 1.877, 95% CI: 1.319–2.669, *p* = 0.00046) were all significantly associated with the discontinuation of TNFis. However, BMI, cigarette smoking, extra-articular manifestations, and patients who use TNFis as their second- or third-line of treatment were not associated with the discontinuation of TNFis. A multivariate analysis identified the predictors of discontinuation as a shorter disease duration (HR: 0.973, 95% CI: 0.948–0.999, *p* = 0.044), and being negative for HLA-B27 (HR: 1.623, 95% CI: 1.127–2.338, *p* = 0.0093, Figure 3). We analyzed the predictors associated with the discontinuation of TNFis focusing only on patients who interrupted their treatment because of inefficacy and on patients using TNFis as first-line treatment; both these sets showed similar data (Supplementary Tables 1–3).

**Table 4:** Predictors of discontinuation of tumor necrosis factor inhibitors.

	**Univariate analysis**				**Multivariate analysis**			
**Variable**	**HR**	**95% CI**		***p*-value**	**HR**	**95% CI**		***p*-value**
Age	1.01030	1.00054	1.02015	0.03851	1.00817	0.99759	1.01887	0.13074
Female gender	1.52504	1.15626	2.01144	0.00281	1.32900	0.94336	1.87229	0.10384
BMI	0.97827	0.94234	1.01556	0.24965				
Disease duration	0.97439	0.95149	0.99784	0.03253	0.97336	0.94817	0.99922	0.04354
Cigarette smoking									
Current	1.11821	0.77314	1.6173	0.55291				
Never	1.19517	0.85197	1.6766	0.30191				
Negative HLA-B27	1.87667	1.31939	2.66931	0.00046	1.62304	1.12673	2.33796	0.00930
Family history of SpA	0.67758	0.42287	1.07930	0.10086				
Peripheral arthritis	1.10825	0.85464	1.43713	0.43818				
Extra-articular manifestation	1.08607	0.84041	1.40354	0.52798				
Syndesmophyte	0.82545	0.62741	1.08600	0.17053				
Biologic use									
Second or more-line users	0.83171	0.62689	1.10347	0.20148				

*BMI, body mass index; HLA, human leukocyte antigen; HR, hazard ratio; CI, confidence interval; SpA, spondyloarthopathy*.

## Discussion

This study demonstrated that discontinuation of TNFis occurred in 24.2% of patients with AS during a median 14-month follow-up period. Few patients with AS who discontinued switched to another TNFis during the treatment course. The most common reason for discontinuation was lack of efficacy (32.6%), followed by adverse events (23.6%), clinical improvement (11.2%), and others (32.6%). The drug retention rate for golimumab was higher compared to other agents prescribed to Korean patients with AS. The predictors of TNFis discontinuation were a shorter disease duration and being negative for HLA-B27.

Data from national registries on the post-marketing use of several biologic agents in the treatment of rheumatic diseases have provided additional information, for example on efficacy, long-term safety, economic data, and real-world trends (18). Although RCTs and open-label studies provide a large amount of data, there is an inherent limitation in that they lack generalizability because they include well-controlled subjects. Therefore, data reflecting the prescription environment of each country obtained from national registries are important. In this regard, KOBIO was designed as a nationwide, multicenter (38 institutes), hospital-based, observation registry in Korea beginning in 2012. The results obtained using KOBIO data can be interpreted as reflecting the national health insurance policy and real clinical practice of physicians in Korea, and provide valuable data for the rheumatology community.

Drug persistence is a reliable tool for assessing drug effectiveness and is affected positively by persistent efficacy and negatively by lack of efficacy and adverse events, for example, as well as by medical and non-medical environmental factors such as insurance policy or coverage and social bias regarding certain treatments (18, 19). An Italian multicenter observational cohort study compared drug survival in different first-line TNFis in 1,220 patients with SpA and 2,640 patients with RA (18). The median follow-up period was 17 months. Drug persistence was slightly better in patients with SpA than in patients with RA, with an HR of 0.83. In particular, among the patients with SpA, those patients with AS showed the lowest discontinuation rate due to lack of efficacy and adverse events. A recent study reported treatment response and drug retention rates in 24,915 biologic-naïve patients with axial SpA initiating TNFis treatment from 12 registries in the EuroSpA collaboration (20). The study showed that 12-month retention rate was 80% ranging from 71 to 94% across registries. Fabbroni et al. (21) evaluated treatment persistence rates to identify the causes of TNFi discontinuation in patients with AS and in patients with PsA. The patients were treated with adalimumab, etanercept, or infliximab with 84.7% of them maintaining their TNFis therapy during more than 6 months of follow-up. The present study evaluated the drug retention rate and reasons for discontinuation in 1,005 patients with AS from the KOBIO registry. In this study, TNFis treatment was not limited to first-line therapy. The median follow-up period was 14 months, with the overall drug retention rate for TNFis being 75.8% in patients with AS. The most common reason for discontinuation was lack of efficacy (32.6%), with the discontinuation rate because of clinical improvement being just 11.2%. These results were similar to previous studies with a similar median follow-up period (18, 21). The results in the current study therefore suggest that drug persistence in Korean patients with AS is comparable to that reported in the literature.

The TNFis investigated in the current study were etanercept, infliximab originator, infliximab biosimilar, adalimumab, and golimumab. The discontinuation rate was found to range from 13.8 to 39.6% among the TNFis. The drug retention rate for golimumab (82.2%) was highest, followed by adalimumab, infliximab biosimilar, etanercept, and infliximab originator. The rate of adverse events decreased to 9.7%, and the rate of significant clinical improvement also dropped to 6.5% for golimumab compared to another TNFi, although the inefficacy rate was similar among the TNFis from etanercept to golimumab ranging from 27.8 to 45.2%. A recent observational study in Italy on golimumab evaluated real-life retention rates in 416 patients with RA, PsA, and AS (22). It showed a global 2-year drug retention rate of 70.2% without differences in discontinuation risks among the diseases. A different Italian study conducted at the same time showed that the 2-year retention rate in 120 patients with AS was 62.8% when given as first- or second-line therapy with similar discontinuation rates including adverse events among the TNFis (23). Our study had a relatively short median follow-up period and showed a high golimumab retention rate compared to previous studies. This result appears to reflect an issue in Korea's policy of TNFis use in patients with AS. In Korea, golimumab and infliximab biosimilar have recently been introduced for the treatment of AS. A change from infliximab originator to infliximab biosimilar may occur for no specific reason. However, changes to other TNFis can only be allowed if the original TNFis has side effects, or is ineffective; in this case, once the TNFis has been changed because of side effects or inefficacy, it cannot be changed back to the original TNFis. The number of patients registered as using golimumab as a first-line TNFis in the current study was lower than that for other TNFis. However, third-line or above TNFis users had a higher rate of golimumab use compared to other TNFis. There are few alternative TNFis for third-line or above use; therefore, physicians tend to be more reticent to switch TNFis than previously. Moreover, in the case of golimumab, subcutaneous injection is administered at an interval of 4 weeks. Patients with AS treated with TNFis are required to visit the hospital every 4 weeks for a period of 6 months starting from 5 years ago as part of their national health insurance policy. It can be assumed that this also contributes to maintenance of the drug retention rate for golimumab.

In the current study, the predictors associated with discontinuation of TNFis were age (HR: 1.010, *p* = 0.03851), female sex (HR: 1.525, *p* = 0.00281), disease duration (HR: 0.974, *p* = 0.03253), and being negative for HLA-B27 (HR: 1.877, *p* = 0.00046) by univariate analysis. A multivariate analysis identified shorter disease duration (HR: 0.973, *p* = 0.044) and being negative for HLA-B27 (HR: 1.623, *p* = 0.0093) as predictors of discontinuation. One study analyzed the baseline predictors of discontinuation of TNFis in 220 patients with AS (24). The predictors associated with discontinuation were female sex, absence of peripheral arthritis, higher BASDAI, and lower erythrocyte sedimentation rate (ESR), or CRP levels. A recent study evaluated the 8-year survival of the initial TNFis in patients with axial SpA and PsA, and identified the predictive factors for discontinuation (25). A high baseline BASDAI (HR: 0.9842, *p* = 0.008) was found to be predictive for a low risk of discontinuation in patients with axial SpA. The predictors identified in previous studies were clinical objective variables, such as high baseline BASDAI, but the predictors in our study were disease duration and negative HLA-B27. These differences may be explained by the different follow-up durations and national insurance guidelines, and may depend on whether only first-line users of TNFis were enrolled.

This study is limited by its observational, non-randomized design, and relatively short duration of follow-up. There are no data on the etanercept biosimilar or the adalimumab biosimilar. Moreover, since this study was an open, non-randomized study, there may also have been a selection bias. Furthermore, it should be noted that there was no washout period in patients who switched TNFis. However, the KOBIO database has been constructed using data from a real-life cohort of biologic users, and so the current study reports the drug retention rate of individual TNFis and identifies predictors for discontinuation of TNFis in Korean patients with AS.

## Conclusion

Our study shows that drug persistence in Korean patients with AS is comparable to that reported in the literature, and that few patients switch to other TNFis during their treatment course. Discontinuation rates between agents may partly be affected by the time of approval by the Korea Food and Drug Administration and the particular national health insurance policy. Follow-up studies with an extended observational period are needed.

## Data Availability Statement

The raw data supporting the conclusions of this article will be made available by the authors, without undue reservation.

## Ethics Statement

KOBIO study was approved by the Institutional Review Board of each participating hospital and informed consent was obtained from all enrolled patients. Current study was approved by the institutional review board of Ajou University Hospital (AJIRB-MED-SUR-12-357).

## Author Contributions

H-AK, S-KL, SO, EP, Y-BP, and KS made a substantial contribution to the conception or design of the study. All authors made a substantial contribution to the acquisition, analysis, and interpretation of the data and manuscript development, and gave final approval of the manuscript for submission.

## Conflict of Interest

The authors declare that the research was conducted in the absence of any commercial or financial relationships that could be construed as a potential conflict of interest.
