# Supplementary material for: Comparison of Retention Rates Between Tumor Necrosis Factor-α Inhibitors in Patients With Ankylosing Spondylitis: Data From the Korean College of Rheumatology Biologics Registry
Source: Front Med (Lausanne). 2021 Jun 15;8:689609. doi: 10.3389/fmed.2021.689609 (PMC8239349; doi:10.3389/fmed.2021.689609)
Supplement: Supplementary file 1 [file Table_1.docx]

Table 1. Baseline characteristics of the study patients

|  | Etanercept | Infliximab  originator | Infliximab  biosimilar | Adalimumab | Golimumab | Total |
| --- | --- | --- | --- | --- | --- | --- |
| N | 148 | 91 | 166 | 375 | 225 | **1005** |
| Age (y) | 41.2 ± 12.8 | 43.0 ± 14.8 | 41.1 ± 12.6 | 40.1 ± 13.0 | 40.3 ± 10.8 | 40.7 ± 12.6 |
| Gender, male, N (%) | 125 (84.5) | 60 (65.9) | 119 (71.7) | 293 (78.1) | 181 (80.4) | **778 (77.4)** |
| BMI | 23.4 ± 3.9 | 22.9 ± 3.2 | 23.9 ± 3.1 | 23.3 ± 3.6 | 23.2 ± 3.2 | **23.4 ± 3.4** |
| Disease duration, yrs | 7.7 ± 6.4 | 6.6 ± 5.3 | 6.1 ± 5.5 | 6.8 ± 5.6 | 8.3 ± 6.5 | **7.1 ± 5.9** |
| Cigarette smoking, n (%) |  |  |  |  |  |  |
| Ex | 37 (25.0) | 18 (19.8) | 32 (19.3) | 77 (20.5) | 46 (20.4) | **210 (20.9)** |
| Current | 50 (33.8) | 25 (27.5) | 45 (27.1) | 110 (29.3) | 72 (32.0) | **302 (30.0)** |
| Never | 61 (41.2) | 48 (52.7) | 89 (53.6) | 188 (50.1) | 107 (47.6) | **493 (49.1)** |
| HLA-B27 positivity, % | 86.5 | 86.8 | 75.3 | 85.9 | 77.3 | **82.4** |
| Family history for SpA, % | 12.2 | 13.2 | 12.7 | 7.2 | 13.3 | **10.8** |
| BASDAI | 6.0 ± 2.0 | 5.4 ± 2.0 | 6.2 ± 1.8 | 5.8 ± 2.0 | 6.3 ± 1.8 | **6.0 ± 2.0** |
| BASFI | 3.5 ± 2.6 | 2.9 ± 2.5 | 3.5 ± 2.4 | 3.5 ± 2.7 | 3.6 ± 2.5 | **3.5 ± 2.6** |
| CRP, mg/dL | 2.6 ± 3.2 | 2.1 ± 3.3 | 2.1 ± 2.6 | 2.1 ± 2.7 | 2.1 ± 2.4 | **2.2 ± 2.7** |
| ASDAS-CRP | 3.7 ± 1.1 | 3.1 ± 1.2 | 3.7 ± 1.0 | 3.5 ± 1.1 | 3.7 ± 1.0 | **3.6 ± 1.1** |
| Patients with syndesmophyte, % | 33.1 | 30.8 | 33.7 | 30.9 | 37.8 | **33.2** |
| Biologic use, N (%) |  |  |  |  |  |  |
| First-line users | 113 (76.4) | 61 (67.0) | 144 (86.8) | 291 (77.6) | 155 (68.9) | **764 (76.0)** |
| Second-line users | 30 (20.3) | 26 (28.6) | 17 (10.2) | 71 (18.9) | 36 (16.0) | **180 (17.9)** |
| Third or more-line users | 5 (3.4) | 4 (4.4) | 5 (3.0) | 13 (3.5) | 34 (15.1) | **61 (6.1)** |

BMI, body mass index; HLA, human leukocyte antigen; SpA, spondyloarthopathy; BASDAI, Bath Ankylosing Spondylitis Disease Index; Bath Ankylosing Spondylitis Functional Index; CRP, C-reactive protein; ASDAS, ankylosing spondylitis disease activity score.

Table 2. Retention rate of individual tumor necrosis factor inhibitors

|  | Etanercept | Infliximab  originator | Infliximab  biosimilar | Adalimumab | Golimumab | Total |
| --- | --- | --- | --- | --- | --- | --- |
|  | (n = 148) | (n = 91) | (n = 166) | (n = 375) | (n = 225) | (n = 1,005) |
| Discontinued, n | 42 | 36 | 42 | 92 | 31 | 243 |
| Discontinuation rate, % | 28.4 | 39.6 | 25.3 | 24.5 | 13.8 | 24.2 |
| Switched, n | 17 | 15 | 24 | 31 | 9 | 96 |
| Switching rate, % | 11.5 | 16.5 | 14.5 | 8.3 | 4.0 | 9.6 |
| Follow-up period, months |  |  |  |  |  |  |
| Min | 0.7 | 0 | 0.5 | 0.4 | 1 | 0 |
| Max | 38 | 39 | 31 | 39 | 34 | 39 |
| Mean | 15.5 | 15.5 | 15.1 | 16.0 | 16.5 | 15.9 |
| Median | 14 | 14 | 13 | 14 | 15 | 14 |

Table 3. Reasons for discontinuation

|  | Total | Etanercept | Infliximab originator | Infliximab biosimilar | Adalimumab | Golimumab |
| --- | --- | --- | --- | --- | --- | --- |
| N | 1,005 | 148 | 91 | 166 | 375 | 225 |
| Discontinuation, n (%) | 243 (24.2) | 42 (28.4) | 36 (39.6) | 42 (25.3) | 92 (24.5) | 31 (13.8) |
| Significant clinical  improvement, n (%) | 27 (11.2) | 3 (7.14) | 3 (8.3) | 5 (11.9) | 14 (15.2) | 2 (6.5) |
| Inefficacy, n (%) | 79 (32.6) | 14 (33.3) | 10 (27.8) | 19 (45.2) | 26 (28.3) | 10 (32.3) |
| Adverse event, n (%) | 57 (23.6) | 14 (33.3) | 12 (33.3) | 9 (21.4) | 19 (20.7) | 3 (9.7) |
| Other reason*, n (%) | 80 (32.6) | 11 (26.2) | 11 (30.6) | 8 (19.1) | 33 (35.9) | 16 (51.6) |

*Includes follow-up loss, planned or confirmed pregnancy, costs or reimbursement issues.

Table 4. Predictors of discontinuation of tumor necrosis factor inhibitors

|  | Univariate analysis | | | | Multivariate analysis | | | |
| --- | --- | --- | --- | --- | --- | --- | --- | --- |
| Variable | HR | 95% CI | | p-value | HR | 95% CI | | p-value |
| Age | 1.01030 | 1.00054 | 1.02015 | **0.03851** | 1.00817 | 0.99759 | 1.01887 | 0.13074 |
| Female gender | 1.52504 | 1.15626 | 2.01144 | **0.00281** | 1.32900 | 0.94336 | 1.87229 | 0.10384 |
| BMI | 0.97827 | 0.94234 | 1.01556 | 0.24965 |  |  |  |  |
| Disease duration | 0.97439 | 0.95149 | 0.99784 | **0.03253** | 0.97336 | 0.94817 | 0.99922 | **0.04354** |
| Cigarette smoking |  |  |  |  |  |  |  |  |
| Current | 1.11821 | 0.77314 | 1.6173 | 0.55291 |  |  |  |  |
| Never | 1.19517 | 0.85197 | 1.6766 | 0.30191 |  |  |  |  |
| Negative HLA-B27 | 1.87667 | 1.31939 | 2.66931 | **0.00046** | 1.62304 | 1.12673 | 2.33796 | **0.00930** |
| Family history of SpA | 0.67758 | 0.42287 | 1.07930 | 0.10086 |  |  |  |  |
| Peripheral arthritis | 1.10825 | 0.85464 | 1.43713 | 0.43818 |  |  |  |  |
| Extra-articular manifestation | 1.08607 | 0.84041 | 1.40354 | 0.52798 |  |  |  |  |
| Syndesmophyte | 0.82545 | 0.62741 | 1.08600 | 0.17053 |  |  |  |  |
| Biologic use |  |  |  |  |  |  |  |  |
| Second or more-line users | 0.83171 | 0.62689 | 1.10347 | 0.20148 |  |  |  |  |

BMI, body mass index; HLA, human leukocyte antigen; HR, hazard ratio; CI, confidence interval; SpA, spondyloarthopathy.
